# Decompressive craniectomy for acute ischemic stroke

**DOI:** 10.1186/s13054-019-2490-x

**Published:** 2019-06-07

**Authors:** Thomas Beez, Christopher Munoz-Bendix, Hans-Jakob Steiger, Kerim Beseoglu

**Affiliations:** Department of Neurosurgery, Medical Faculty, Heinrich-Heine-University, Moorenstrasse 5, 40225 Düsseldorf, Germany

**Keywords:** Decompressive craniectomy, Hemicraniectomy, Suboccipital craniectomy, Cranioplasty, Malignant ischemic infarction, Pediatric stroke

## Abstract

Malignant stroke occurs in a subgroup of patients suffering from ischemic cerebral infarction and is characterized by neurological deterioration due to progressive edema, raised intracranial pressure, and cerebral herniation. Decompressive craniectomy (DC) is a surgical technique aiming to open the “closed box” represented by the non-expandable skull in cases of refractory intracranial hypertension. It is a valuable modality in the armamentarium to treat patients with malignant stroke: the life-saving effect has been proven for both supratentorial and infratentorial DC in virtually all age groups. This leaves physicians with the difficult task to decide who will require early or preemptive surgery and who might benefit from postponing surgery until clear evidence of deterioration evolves. Together with the patient’s relatives, physicians also have to ascertain whether the patient will have acceptable disability and quality of life in his or her presumed perception, based on preoperative predictions. This complex decision-making process can only be managed with interdisciplinary efforts and should be supported by continued research in the age of personalized medicine.

## Background

### Introduction

Primary insults to the brain can lead to cerebral edema and intracranial hypertension, which are major mechanisms of secondary brain damage and thus significant determinants of mortality and poor outcome. With the advent of modern neurosurgery and critical care, the old technique of decompressive craniectomy (DC), i.e., surgically opening the skull to relieve raised intracranial pressure (ICP), was refined and put into the focus of clinical research especially in the fields of traumatic brain injury (TBI) and ischemic cerebral infarction. This review will provide detailed insight into the history and evidence base of DC for acute ischemic stroke, the status quo of this treatment option in modern interdisciplinary stroke care, and a stimulating future perspective.

### History

**Fig. 1:**
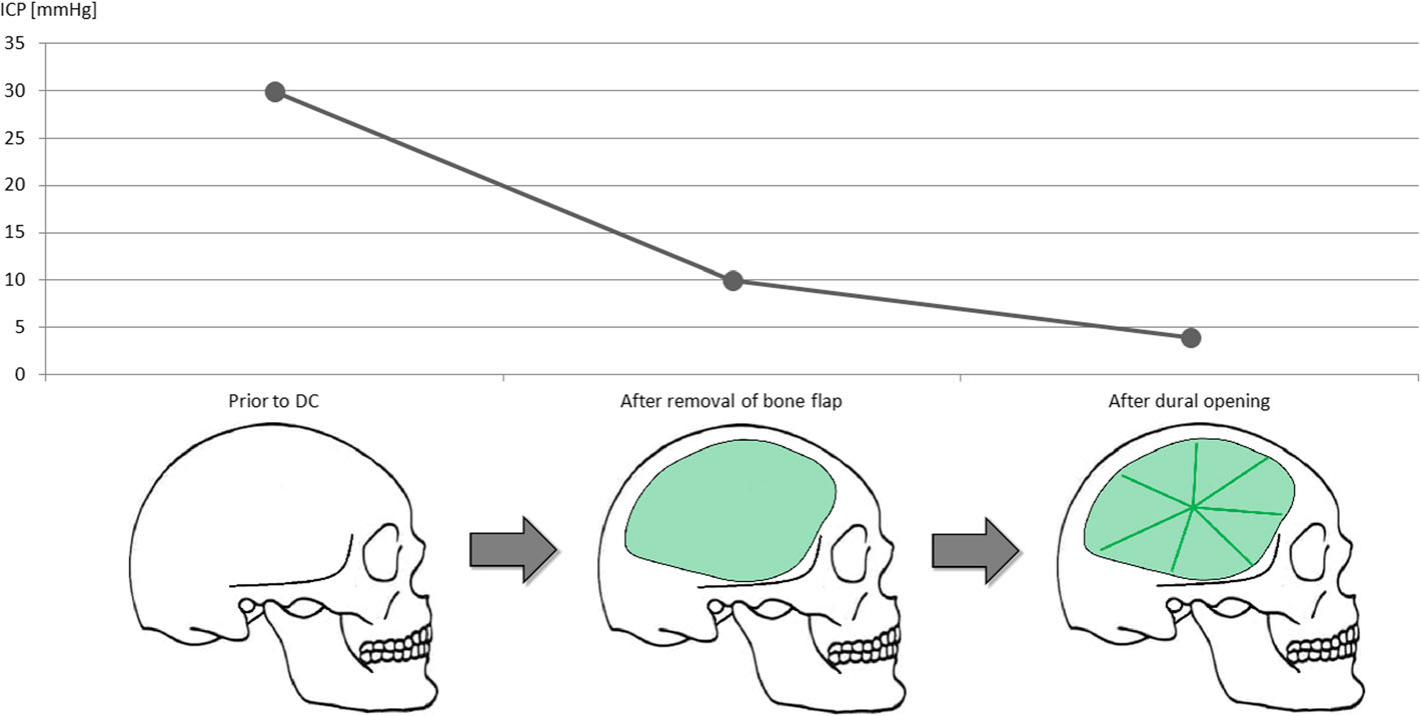
Stepwise reduction of ICP during DC. Representative ICP measurements obtained during DC performed on an 11-year-old boy suffering from refractory intracranial hypertension. Removal of the bone flap reduces ICP by 66% from 30 to 10 mmHg, followed by a further 50% reduction to 5 mmHg after dural opening

Trephination is the earliest technique of opening the skull and can be traced back to at least 12,000 years before Christ, indicated by the discovery of primitive surgical tools, corresponding skull defects, evidence of bone healing, or even cranioplasty on human skulls [1]. Whether such ancient operations were performed to treat TBI or as part of religious rituals remains a historical mystery. However, the earliest pathophysiological concepts and surgical techniques resembling our modern understanding of DC were published at the beginning of the twentieth century. In 1901, Theodor Kocher stated that “pressure relieve by surgical trepanation is clearly indicated in all cases of intracranial hypertension” [2]. Although he primarily referred to TBI, he further elaborates that “time to act has come in any case of brain damage leading to progressive and severe neurological impairment”. In 1908, Harvey Cushing precisely described primary and secondary brain injury and thus paved the way for his concept of subtemporal DC for TBI [3]: “[…] the symptoms of most of these cases are brought about by an increase of intracranial pressure, whether immediate from free extravasation due to the laceration of cortical vessels, intermediate, often with a “free interval” of consciousness, when an extravasation outside of the dura slowly augments in size, or late, often a matter of a few days, when cerebral edema occurs. In many cases, indeed, the symptoms of these various conditions shade imperceptibly into one another. The phenomena of compression are so well understood that they need not be detailed; the slowed pulse, the rise in blood-pressure, the headache, vomiting, and choked disc are seen in their most typical guise in these cases. […]”. While these initial treatises on decompressive operations primarily addressed TBI, the knowledge on ischemic stroke and subsequent edema increased in the following decades and first reports of DC in this field began to emerge in the 1950s [4, 5]. In 1974, Henrique S. Ivamoto and co-workers published a detailed case report of decompressive hemicraniectomy for malignant middle cerebral artery (MCA) infarction [6]. They provided measurements of ICP before, during, and after the operation, indicating a significant alleviating effect of DC on intracranial hypertension (Fig. 1). Additionally, the authors provided the first systematic review of DC for 17 cases of cerebral and 4 cases of cerebellar infarction, concluding that extensive ischemic stroke can cause significant cerebral edema and thus severe pressure effects. However, they highlighted that in the absence of a controlled trial, the benefits of DC for cerebral or cerebellar infarcts are not conclusive. At least for anterior circulation stroke, such randomized controlled trials (RCTs) were finally conducted in the 2000s.

While the entity of cerebellar ischemic stroke was first described in the late 1930s [7], the first case reports of suboccipital decompression were published in 1956 [8, 9]. In 1970, James R. Lehrich and co-workers published a detailed case series of patients with brainstem compression secondary to cerebellar ischemic stroke, along with a literature review, advocating early diagnosis and surgical treatment of this life-threatening condition [10].

### Definitions of malignant cerebral infarction

Occlusion of the internal carotid artery (ICA) or MCA leads to significant cerebral ischemic infarction, accounting for approximately 10% of supratentorial ischemic stroke cases [11]. Hypodensity of more than 50–75% of the MCA territory including the basal ganglia, involvement of additional vascular territories, and cerebral midline shift of more than 4 mm at the level of the pineal gland in the initial 48 h indicate life-threatening infarct volume, i.e., malignant cerebral infarction [12, 13]. Neurological deterioration occurs within 5 days, with the highest frequency of deaths due to transtentorial herniation and subsequent brain death on day 3 after the ictus [14]. The mortality of malignant MCA infarction is around 80% without neurosurgical intervention [15].

The pattern of arterial occlusion found in cerebellar ischemic stroke is more variable. However, 20% of patients suffer from malignant cerebellar stroke with clinical deterioration due to edema, brainstem compression, upward and downward herniation, and occlusive hydrocephalus [16]. In severe cases, bilateral cerebellar infarction, occlusion of the posterior inferior cerebellar artery (PICA), and additional brainstem infarction are typically found [17].

## Technical aspects of DC

### Surgical technique for supratentorial DC

**Fig. 2:**
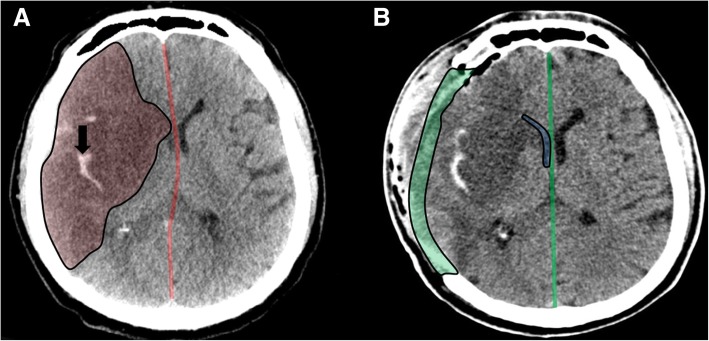
Decompressive hemicraniectomy for malignant ischemic stroke. Axial CT scan before surgery (**a**), demonstrating a demarcated right-sided MCA infarct (highlighted in red) with hemorrhagic transformation (black arrow) and midline shift to the left side (red line). Axial CT scan after surgery (**b**), showing the craniectomy defect (highlighted in green) with decompressed lateral ventricle (highlighted in blue) and reversal of midline shift (green line)

**Fig. 3:**
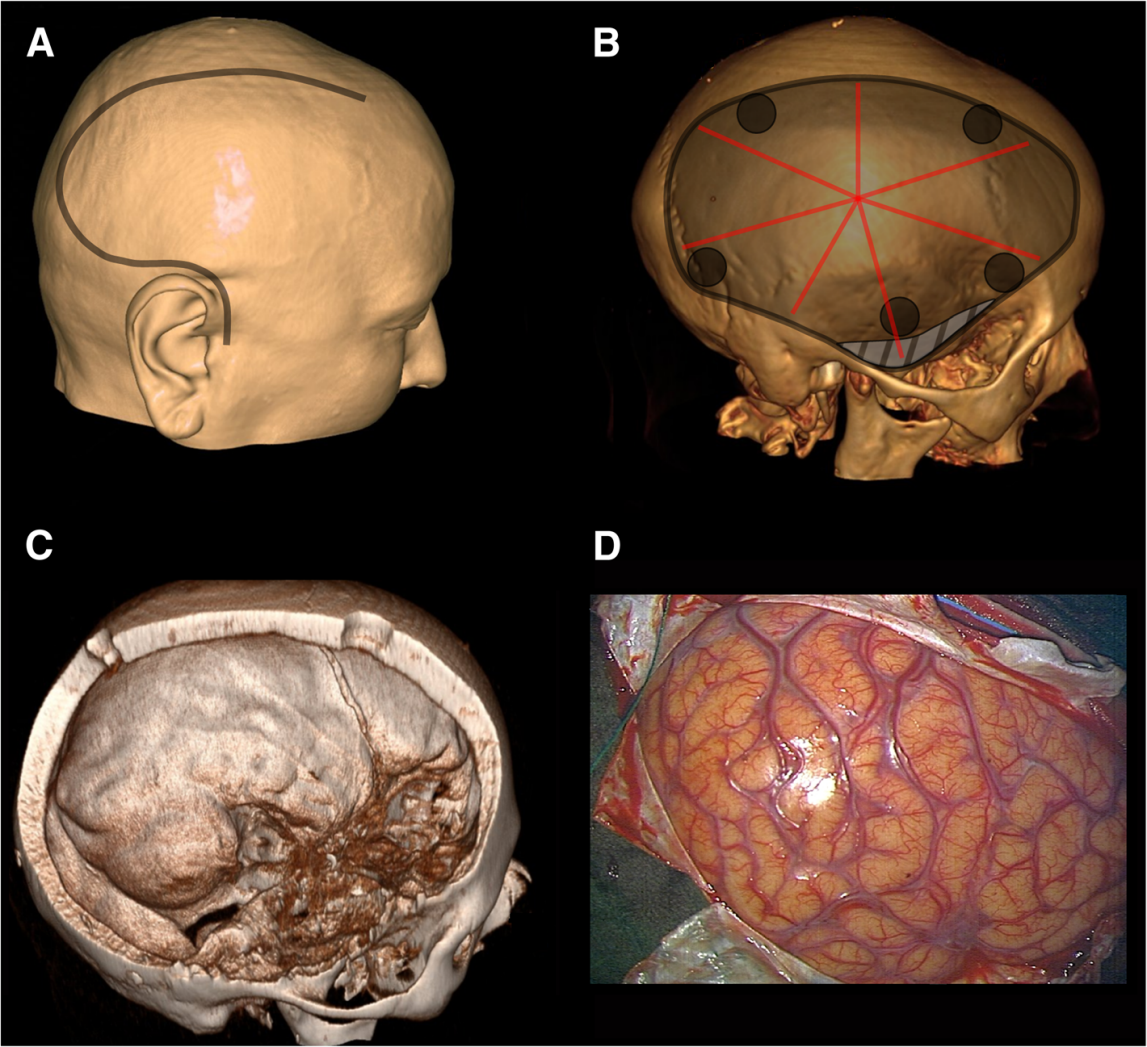
Operative technique of supratentorial DC. Artist’s rendition of a human head (**a**) with a typical incision line for DC (gray line). 3D reconstruction of a human skull (**b**) demonstrating burr holes (gray circles), craniectomy (gray area), and additional osteoclastic decompression of the middle cranial fossa floor (hatched area) as well as typical dural incision (red lines). 3D reconstruction of a human skull (**c**) with a typical hemicraniectomy skull defect. Intraoperative photography of a human brain after DC (**d**)

Unlike traumatic brain injury, ischemic stroke usually affects one cerebral hemisphere, and thus, the surgical aim is decompression of the corresponding area (Fig. 2). Therefore, the typical operation performed in such patients is a fronto-temporo-parietal decompressive hemicraniectomy. While technical details certainly vary between individual surgeons or centers, this brief outline describes a typical operation: the procedure is performed in a supine position with the head rotated to the contralateral side. A wide curved incision is performed either beginning behind or in front of the ear (Fig. 3a). The scalp flap and temporalis muscle are then deflected to expose the skull. Burr holes are created and subsequently connected to achieve an anterior to posterior diameter of the craniectomy area of at least 12 cm, with the recommended diameter in adult TBI patients being 15 cm (Fig. 3b) [18]. The DC is finally extended to expose the floor of the middle cranial fossa (Fig. 3c). An adequately sized craniectomy is essential in achieving the desired decompressive effect. Moreover, a suboptimal DC will lead to exacerbated external brain herniation and shear forces at the bone edges, which can cause intraparenchymal hemorrhage and kinking of the cerebral veins [19].

After sufficient bony decompression has been achieved, the dura is incised to create a large dural opening (Fig. 3b, d). For coverage of the exposed brain, allogenic or autologous dural grafts can be used.

### Surgical technique for infratentorial DC

**Fig. 4:**
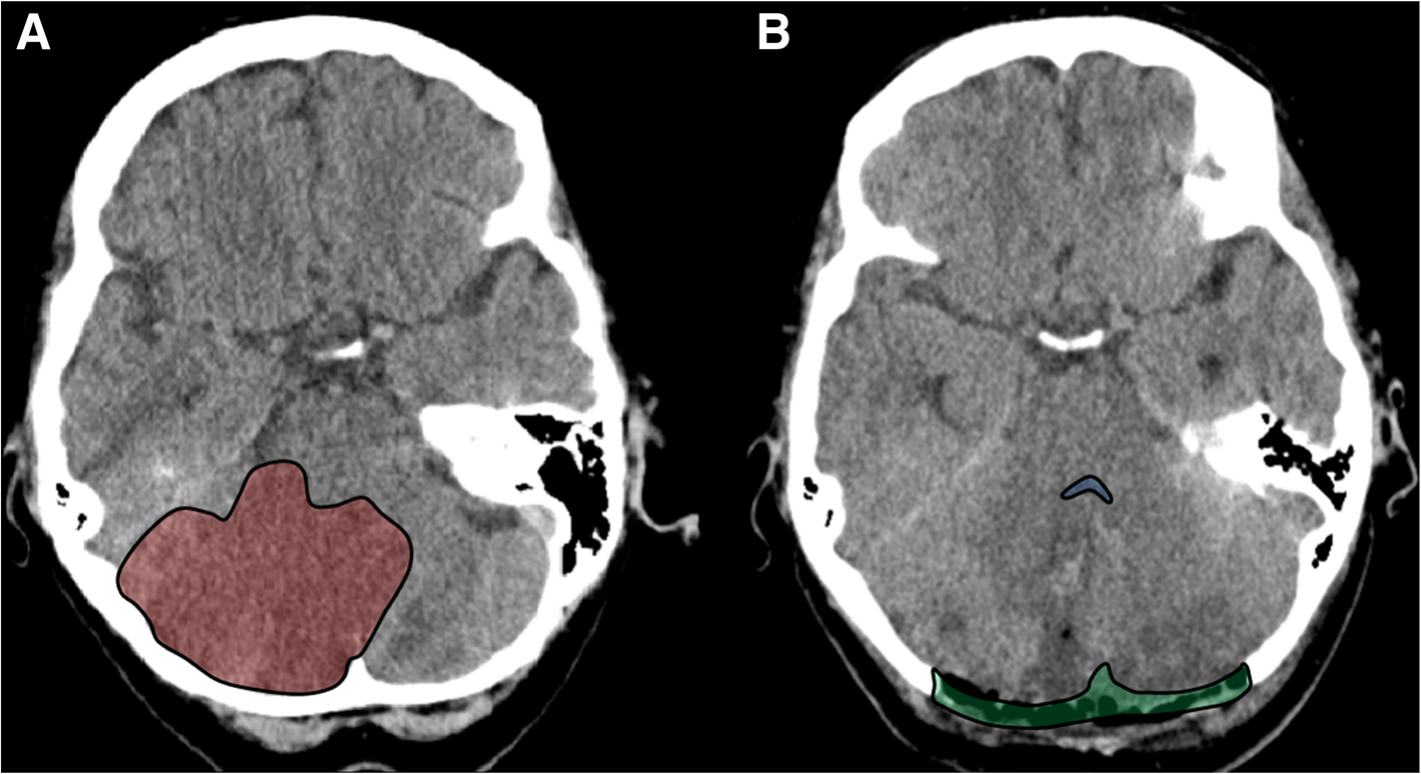
Suboccipital decompressive craniectomy for malignant cerebellar stroke. Axial CT scan before surgery (**a**), showing a large demarcated cerebellar infarct (highlighted in red). Axial CT scan after surgery (**b**), demonstrating the craniectomy defect (highlighted in green) and decompressed fourth ventricle (highlighted in blue)

In comparison with supratentorial DC, the technical details of suboccipital or infratentorial DC are less clearly established. Important aspects such as overall craniectomy size, laterality of the decompression, and necessity of resection of the posterior arch of the atlas all vary in the published literature. However, the basic surgical aim is decompression above the swollen cerebellum (Fig. 4).

**Fig. 5:**
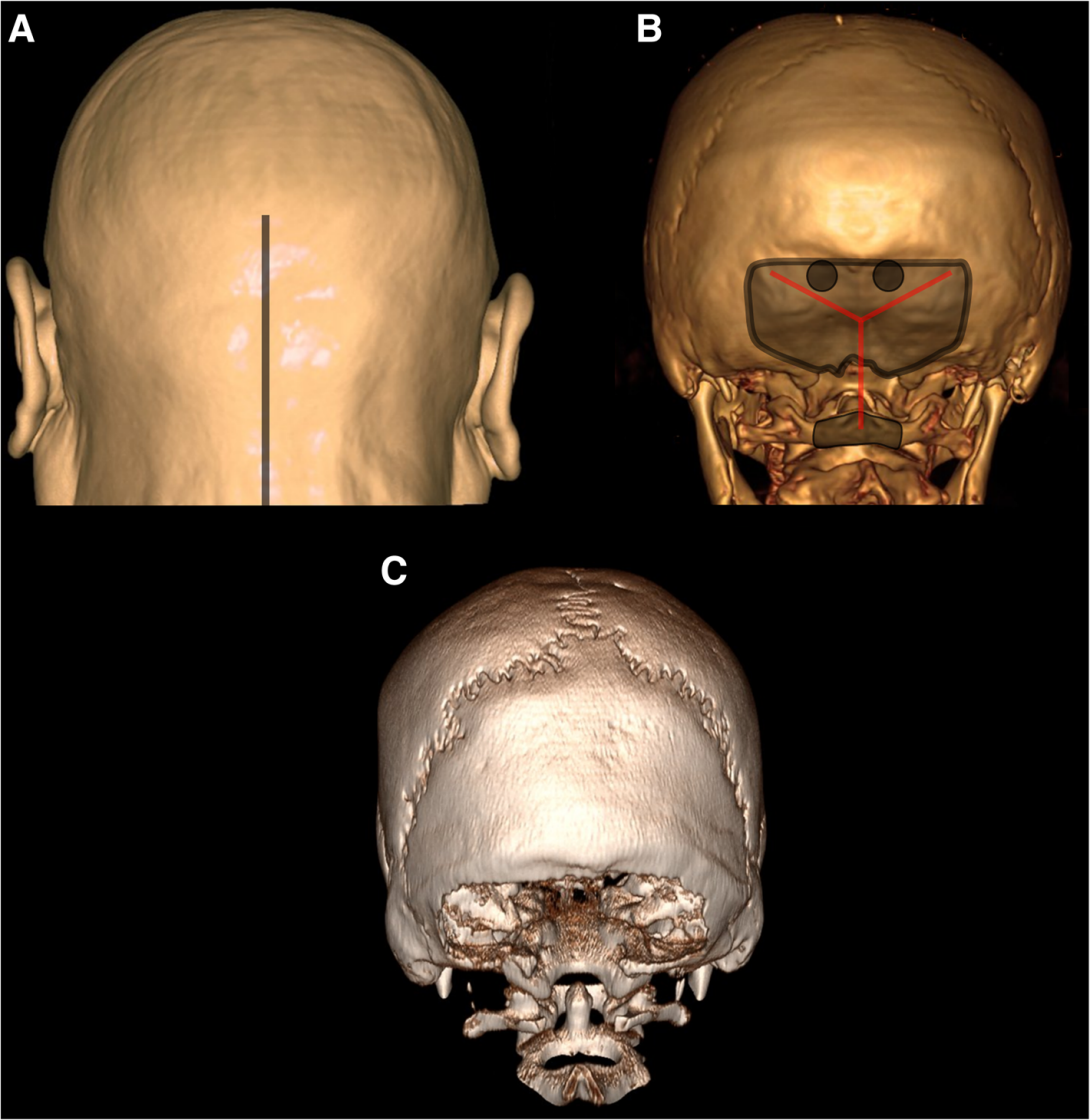
Operative technique of infratentorial DC. Artist’s rendition of a human head (**a**) with a typical incision line for suboccipital DC (gray line). 3D reconstruction of a human skull (**b**) demonstrating burr holes (gray circles), craniectomy, and removal of the posterior arch of the atlas (gray areas) as well as typical dural incision (red lines). 3D reconstruction of a human skull (**c**) with a typical suboccipital DC skull defect

In general, this procedure is performed with the patient in a prone or semi-prone/lateral position. A linear midline incision is made from the inion to the upper cervical spine, and the muscular layers are subsequently separated in the midline avascular plane (Fig. 5a), exposing the suboccipital skull, atlanto-occipital membrane, and posterior arch of the atlas. A wide craniectomy is performed extending into the foramen magnum (Fig. 5b). As the next step, to avoid tonsillar herniation, we routinely remove the posterior arch of the atlas (Fig. 5b). The dura is then usually opened in a Y-shaped fashion, and an expansion duroplasty is performed (Fig. 5c).

### Storage of bone flaps and cranioplasty

After supratentorial DC, the bone flaps are preserved under sterile conditions for autologous cranioplasty at a later stage. The two most common options for bone flap preservation are storage at low temperature (usually − 80 °C or below) or implantation into the patient’s abdominal subcutaneous fat. With both methods being feasible and safe, no evidence-based recommendation can be provided [20].

In patients undergoing suboccipital DC, the bone flaps are not preserved, since cranioplasty is not routinely performed as the craniectomy defect is covered by the neck muscles and no cosmetic deformity or risk of external injury arises.

**Fig. 6:**
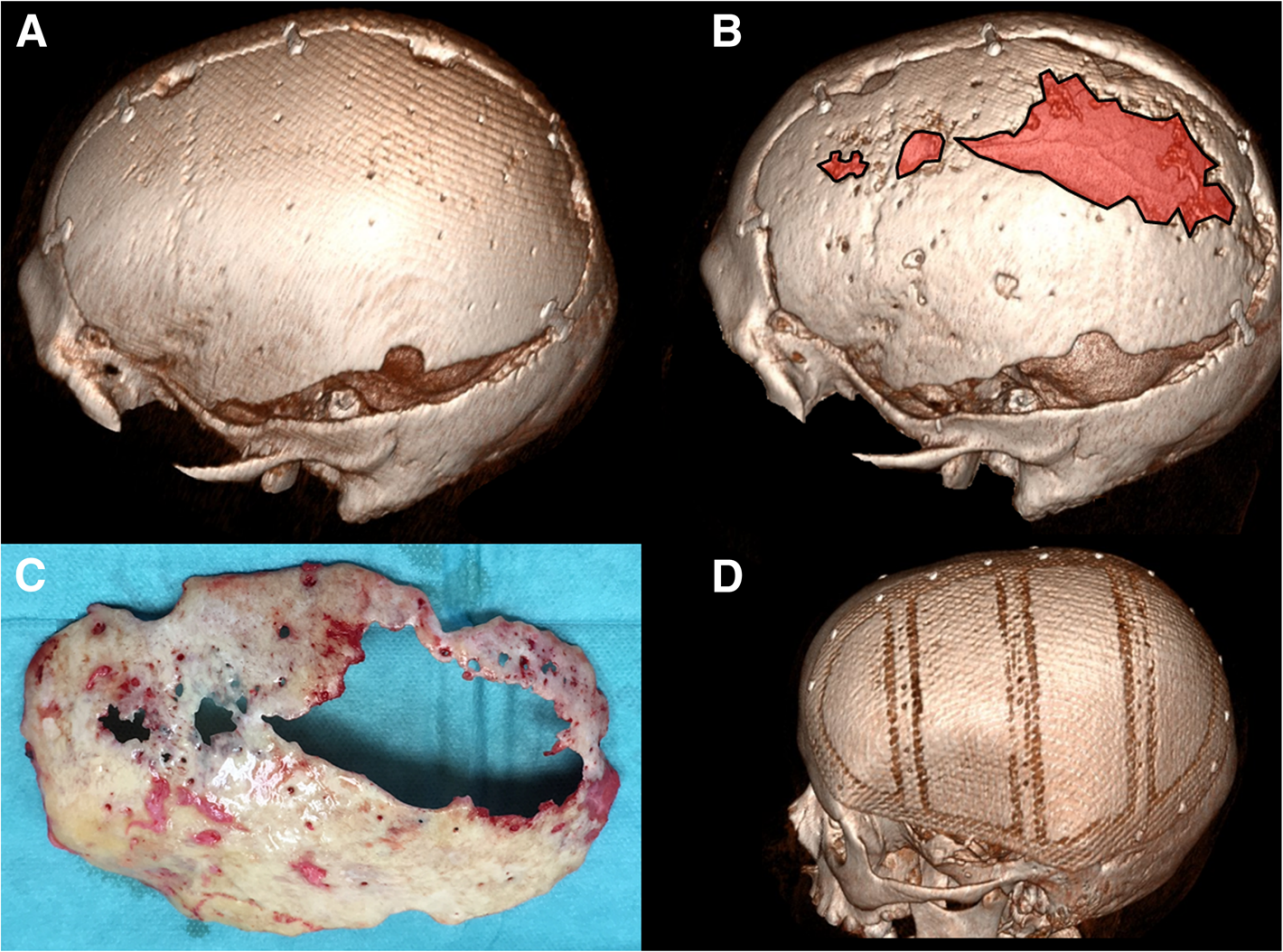
Cranioplasty and autologous bone flap resorption. 3D reconstruction of a 57-year-old male patient’s skull after autologous cranioplasty following DC for left-sided malignant MCA infarction (**a**). One year later (**b**), significant areas of the bone flap resorption occurred (highlighted in red). The explanted autologous bone flap (**c**) shows the overall thinning and obvious holes due to resorption. A typical example of an alloplastic implant (**d**) after right-sided DC in another patient (11-year-old boy after TBI)

While not in the primary focus of this review, cranioplasty is an integral part of surgical treatment for supratentorial stroke and has to be taken into account when making treatment decisions and counseling patients and relatives. The procedure has a relevant complication rate of 30%, with approximately one in four affected patients needing revision surgery [21]. The most common complications are infection, wound breakdown, and postoperative hemorrhage. Early cranioplasty (i.e., within 2 months after DC) appears to be associated with a higher complication rate [22]. A further important determinant of the reoperation rate is the type of cranioplasty: autologous cranioplasty has a significant rate of bone resorption (especially in children) and thus implant failure, often requiring revision surgery with implantation of an alloplastic patient-specific implant (Fig. 6) [23, 24]. At present, no evidence-based recommendation on the use of autologous bone flaps versus alloplastic implants can be given, and the complex interactions between underlying disease, patient age, implant type and preservation method, and timing of cranioplasty are incompletely understood.

## Supratentorial malignant ischemic stroke in adults

### Best available evidence: randomized controlled trials

**Fig. 7:**
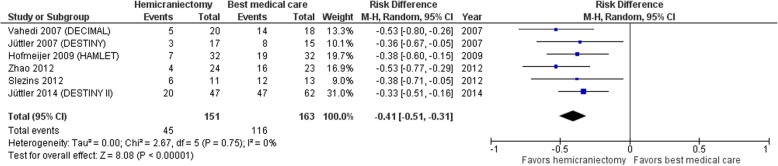
Mortality at 12 months after malignant MCA infarction. Forest plot presenting risk difference and 95% confidence interval (CI) for a pooled analysis of mortality at 12 months from RCTs comparing DC and best medical care

Until today, to the best of our knowledge, the results of eight RCTs investigating the role of DC in malignant MCA stroke are available for analysis, which are summarized in Table 1 [25–32]. Between 2007 and 2009, the results from the first three RCT (DESTINY, DECIMAL, and HAMLET) were published [25–27] and a pooled analysis of patients aged between 18 and 60 years undergoing DC within 48 h after stroke onset was performed [33]. This pooled analysis revealed a significant benefit in all predefined subgroups (e.g., age above and below 50 years, time to randomization above and below 24 h, and dominant versus non-dominant hemisphere) with a number needed to treat of 4 for the prevention of poor outcome (i.e., mRS 4 to 6) and of 2 for survival. In the surgical arm, the probability of survival increased from 30 to 80%, albeit with a tenfold increase in the probability of surviving with a modified Rankin Scale (mRS) score of 4, meaning moderately severe disability requiring assistance from caregivers. However, the probability of surviving with a mRS of ≤ 3 (i.e., slight or moderate disability) doubled and the risk of surviving with a mRS of 5 (i.e., severe disability) remained stable compared to conservative treatment. The pooled analysis of all RCTs providing information on mortality at 12 months follow-up shows a consistent and significant benefit of DC (Fig. 7), with a risk reduction of almost 50%.

**Table 1:** Overview of RCTs investigating the role of DC in malignant MCA infarction. Basic study characteristics were extracted from the corresponding publications. Patient age and timing of randomization or timing of DC after stroke onset are compared between the protocol and actual findings. Information of treatment arms and primary end point is provided

Study	Age [years] (inclusion criteria/actual mean age)	Timing of randomization or DC after stroke onset [h] (protocol/actual mean time)	Treatment arms	Results for good outcome (mRS 0–3) and mortality in surgical vs. conservative arms
Jüttler (DESTINY) [25]	18–60/43.2	< 36/24.4	Hemicraniectomy vs. detailed conservative treatment protocol	47% vs. 27% 18% vs. 53% at 6 months
Vahedi (DECIMAL) [26]	18–55/43.5	< 30/20.5	Hemicraniectomy vs. detailed conservative treatment protocol	25% vs. 6% 25% vs. 78% at 6 months
Hofmeijer (HAMLET) [27]	18–60/50.0	< 96/41	Hemicraniectomy vs. detailed conservative treatment protocol	25% vs. 25% 22% vs. 50% at 12 months
Slezins [28]	≥ 18/57.2	< 48/21	Hemicraniectomy vs. conservative treatment (unspecified)	23% vs. 38% 39% vs. 55% at 12 months
Zhao [29]	18–80/63.5	< 48/23.6	Hemicraniectomy vs. detailed conservative treatment protocol	21% vs. 4% 13% vs. 61% at 6 months
Frank (HeADDFIRST) [30]	18–75/52.3	< 96/53.8	Hemicraniectomy vs. detailed conservative treatment protocol	29% vs. 30% 36% vs. 40% at 6 months
Jüttler (DESTINY II) [31]	≥ 61/70	< 48/28	Hemicraniectomy vs. detailed conservative treatment protocol	7% vs. 3% 33% vs. 70% at 6 months
Chua (HeMMI) [32]	18–65/50.3	< 72/36.6	Hemicraniectomy vs. detailed conservative treatment protocol	23% vs. 38% 39% vs. 55% at 6 months

### Patient age

The main limitation to the generalizability of the RCT results appeared to be age. In 2012, Rahme and co-workers analyzed a large cohort from a population-based epidemiological study in North America with regard to the eligibility for the aforementioned RCTs [34]. Among more than 2000 ischemic stroke cases, less than 1% fulfilled the inclusion criteria, with almost 80% of patients being above the age limit. In subsequent RCTs, the age limit was therefore increased to 80 years or the studies had no age limit at all [28–31]. The DESTINY II trial enrolled patients with a median age of 70 years and was stopped early, as a significant benefit of DC became apparent [31]. Similar to previous RCTs, the survivors had a relevant burden of disability, with 32% of patients having mRS 4 and 19% mRS 5 at 12 months follow-up. However, if data from non-randomized trials is taken into account, the outcome of patients aged 60 years and older appears to be worse than in younger patients [35]. In contrast, an analysis of a large database of Japanese DC patients indicates no age-related differences in the outcome, with 80% above the age of 60 years in this cohort [36].

### Dominant versus non-dominant cerebral hemisphere

As mentioned above, the pooled analysis of DESTINY, DECIMAL, and HAMLET revealed a significant benefit independent of the presence of aphasia [33]. However, crude outcome measures such as mRS might not be adequate to assess the impact of aphasia and its implications for quality of life in detail. Kastrau and co-workers published a thorough assessment of aphasic syndromes after DC for malignant MCA infarction of the dominant hemisphere [37]. They found variable but significant improvement in more than 90% of patients, with younger patients and those treated early faring better.

### Timing of DC

The aim of HAMLET was to assess the impact of DC within 4 days after stroke onset, in contrast to most other RCTs with much shorter time frames (Table 1) [27]. In patients randomized after more than 48 h from the onset of symptoms, no significant benefit with regard to death or poor outcome was found: the absolute risk reduction was only 8%, respectively, comparing to 59% and 30% for patients randomized within 48 h. The pooled analysis of DESTINY, DECIMAL, and HAMLET included only patients treated within 48 h and compared subgroups randomized within versus after 24 h [33]. DC was beneficial in both subgroups, with no significant difference in the outcome.

Dasenbrock and co-workers analyzed a large national database of 1300 patients undergoing DC in routine medical care, i.e., outside clinical trials [38]. They found that 56% of patients underwent DC within 48 h, but worse outcomes were only seen if surgery was performed after more than 72 h from stroke onset. Interestingly, timing appeared to be no significant determinant of outcome in the subset without signs of cerebral herniation. However, a significant interaction between timing, herniation, and outcome was detected, which suggests that performing DC before herniation is the most important temporal factor. This clinical paradigm is supported by pathophysiological concepts, as increasing cerebral edema and raised ICP lead to impaired cerebral perfusion in the non-ischemic parenchyma as part of a cascade known as secondary brain damage [39]. Our group has demonstrated that DC improves the cerebral perfusion in the penumbra, in the residual ipsilateral parenchyma, and in the contralateral hemisphere [40].

Of note, Cho and co-workers published a small retrospective analysis of patients undergoing ultra-early DC within a mean time of 4.25 h after stroke onset [41]. The mortality in this ultra-early group was only 8%, comparing favorably to 38% observed in those treated later, i.e., within a mean time of 68.25 h. While this study demonstrates the technical feasibility of performing such ultra-early operations, the results have to be interpreted with caution. It might be difficult to reliably identify candidates for DC so early after stroke onset, implying that the subgroup might include patients who would not have needed DC according to conventional criteria.

### Monitoring of ICP and secondary interventions

The value of ICP monitoring after DC for ischemic stroke is a matter of ongoing debate. Treatment protocols in RCTs are divergent concerning this aspect, with for instance DESTINY recommending and DECIMAL not recommending invasive ICP monitoring. Sauvigny and co-workers analyzed ICP data after DC for malignant MCA infarction and demonstrated a significant difference in mean ICP values between the subgroups with good (mRS ≤ 4, mean ICP 11.7 mmHg) and poor outcome (mRS 5 + 6, mean ICP 18.7 mmHg) [42]. The therapeutic consequences of elevated ICP can be variable: Paldor and co-workers reported frequent episodes of intracranial hypertension after DC, which were treated with intensive care measures such as drainage of cerebrospinal fluid, modification of sedation, hyperosmotic therapy, cooling, head elevation, and moderate hyperventilation [43]. In the studies of Schwake and co-workers as well as Kürten and co-workers raised ICP after DC even triggered secondary debridement of infarcted tissue, and this intervention was found to at least reduce case fatality rates [44, 45].

### Surgical complications of DC

Surgical complications of DC can occur at any stage but are commonly classified into early (i.e., during the initial admission for acute stroke) and late complications (in the subacute and recovery phases). They should be differentiated from cerebral herniation and brain death occurring in patients after DC, as this is usually a result of the insult itself rather than associated with treatment. From a pathophysiological point of view, surgical complications after DC mainly relate to hemorrhage, infection, cerebrospinal fluid disturbance, and seizures. The reported rates appear to be lower in RCTs (Table 2) compared to the overall literature, as summarized by Kurland and co-workers [46]. In comparison with surgical complications, general complications (such as pneumonia, urinary tract infections, and venous thrombosis) are more common [26].

**Table 2:** Surgical complications after supratentorial DC. Data on surgical complications was extracted from publications of RCTs. Comparable information was found in only three articles, and representative percentages for the most relevant complications were calculated

RCT	No. of patients in surgical arm	Hemorrhage	Central nervous system and surgical site infection	CSF leaks, hygroma, and hydrocephalus	Seizures
Vahedi (DECIMAL) [33]	20	0	1	0	7
Hofmeijer (HAMLET) [27]	32	1	0	2	1
Chua (HeMMI) [32]	13	1	1	0	0
	65	2 (3%)	2 (3%)	2 (3%)	8 (12%)

Malignant stroke patients can be considered a high-risk group for hemorrhagic complications, as 40% are on prior antiplatelet medication and 45% receive intravenous thrombolytic therapy [47]. Approximately 10% of patients will have at least radiological evidence of epidural hemorrhage after DC, with only a fraction requiring revision surgery [46]. Hemorrhagic transformation of ischemic stroke occurs in 30 to 60%, with a large proportion being present already before DC (Fig. 2) [47, 48]. Antiplatelet therapy but not intravenous thrombolysis appears to be a risk factor for perioperative hemorrhage [47]. Intra-arterial thrombolysis and thrombectomy prior to DC do not appear to increase the risk of hemorrhage [49].

Infections of the surgical site or within the central nervous system are observed in less than 10% of patients after DC, including wound infections, empyema, and cerebral abscess [46]. In RCT, such infectious complications were rare and comprised superficial wound infection and cerebral abscess [26, 32]. If an external ventricular drain is inserted for ICP monitoring, the potential risk of ventriculitis will increase over time, especially with prolonged drainage exceeding 1 week [50]. The use of antibiotic-impregnated ventricular catheters can minimize this risk to less than 5% [51].

**Fig. 8:**
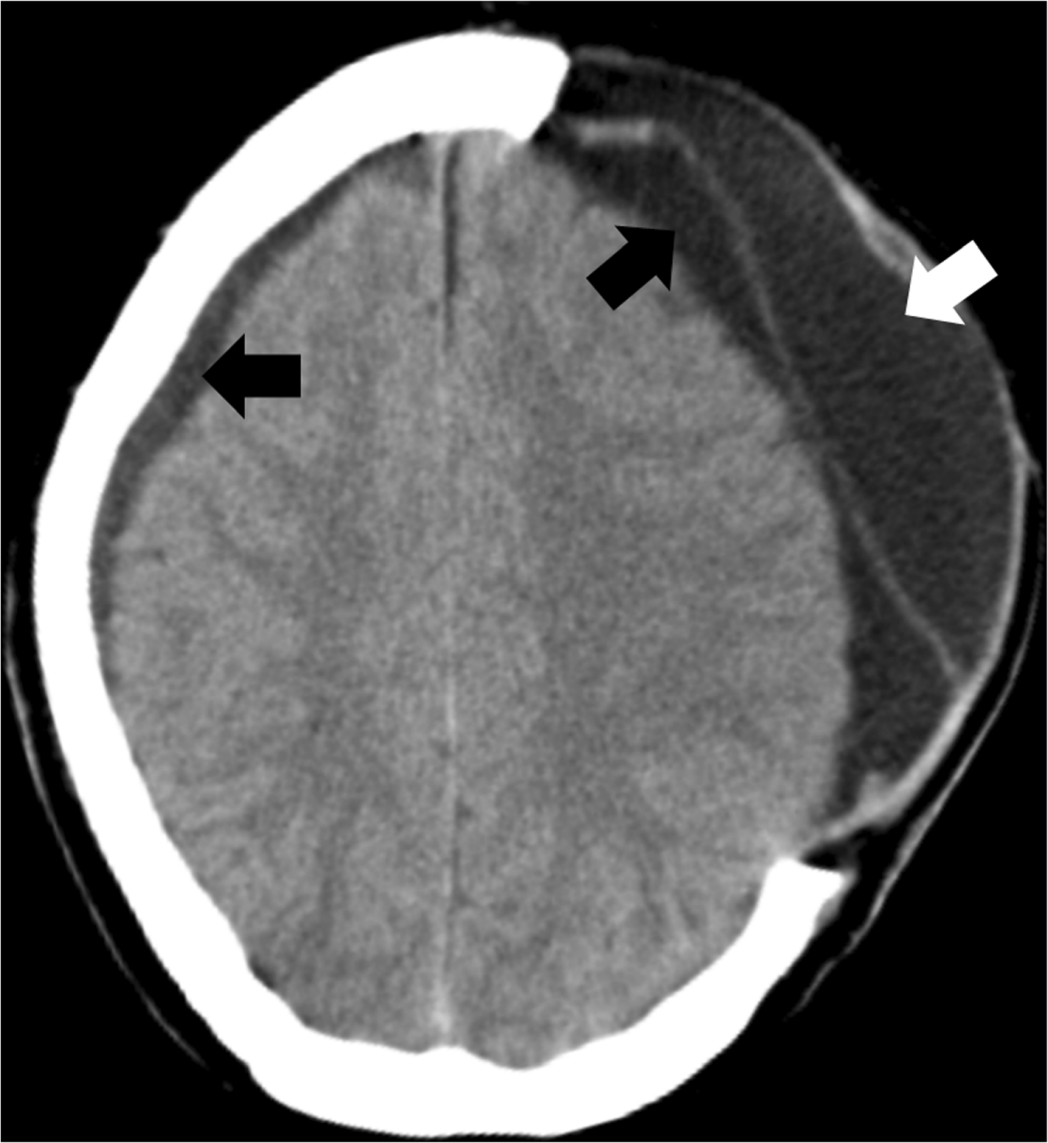
Hygroma occurring after DC. Axial CT scan showing ipsilateral and contralateral subdural hygroma (black arrows), which occurred after left-sided hemicraniectomy. Additionally, a large epidural fluid collection (white arrow) is observed

Cerebrospinal fluid (CSF) disturbances are frequently encountered after DC for cerebral infarction: 20 to 80% develop hygroma and 30 to 40% internal communicating hydrocephalus (Fig. 8) [52–54]. At least one third of these CSF disturbances appear to resolve either spontaneously or after cranioplasty. In the remaining patients, a ventriculo- and/or subduro-peritoneal shunt might be required to avoid further neurological deterioration.

Population-based studies of seizures in stroke survivors have demonstrated a 6 to 12% risk of single or recurrent seizures within 5 years [55, 56]. Anterior circulation stroke and stroke severity are predictors of increased seizure risk. Thus, patients qualifying for DC after malignant cerebral infarction are per se a high-risk group: 50% suffer a seizure and 45% will develop epilepsy [57]. Importantly, the authors found a clustering of first seizures within weeks after cranioplasty, indicating cranioplasty rather than DC itself as a major risk factor.

**Fig. 9:**
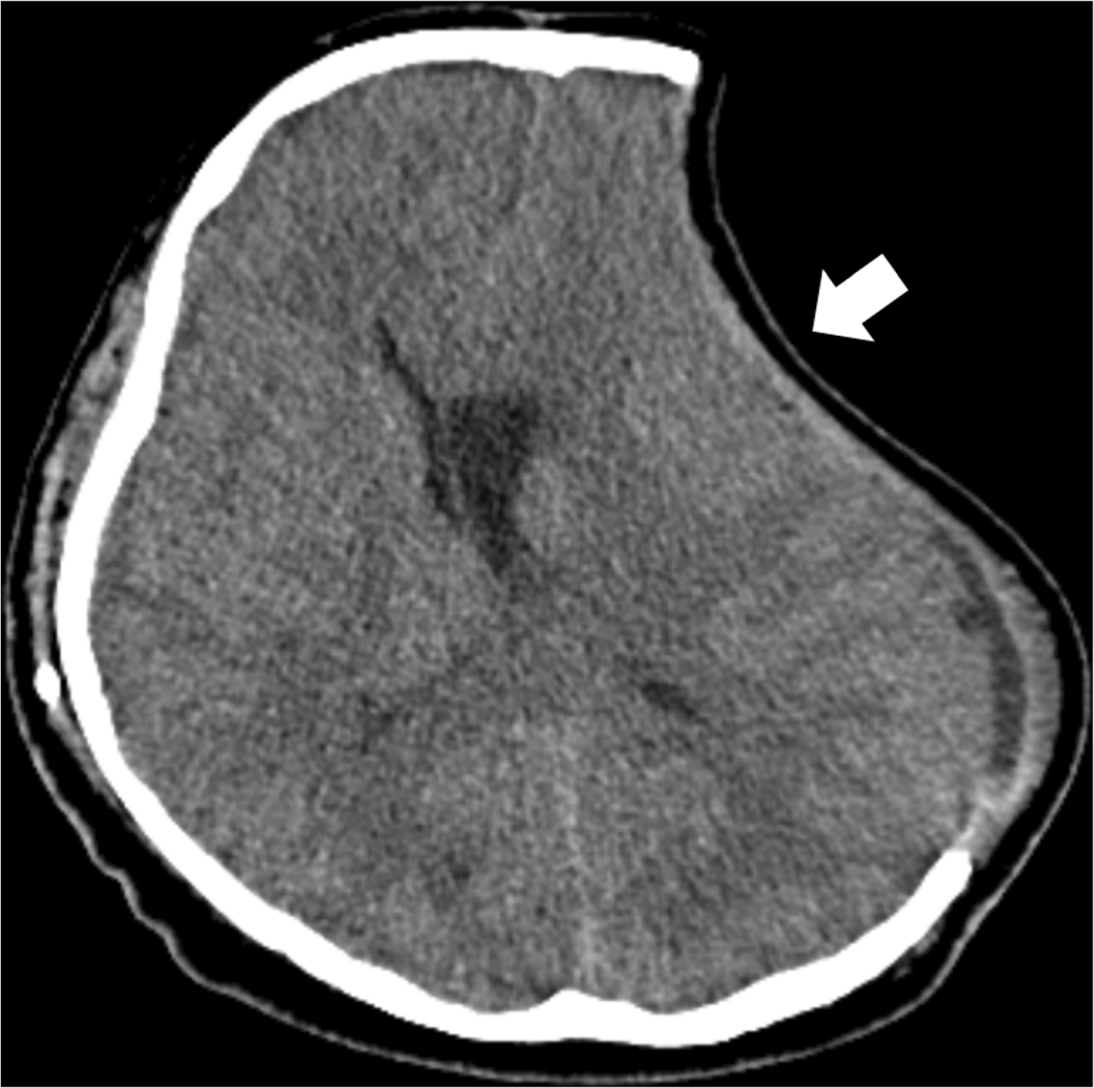
Sunken skin flap after DC. Axial CT scan showing a sunken skin flap (white arrow) after left-sided hemicraniectomy

A long-term complication after DC is the syndrome of the trephined or sunken skin flap syndrome, which occurs weeks to months after DC and is often characterized by neurological deterioration after initial rehabilitative improvements [58]. An obvious finding on clinical and radiological examinations of affected patients is the severely sunken sink flap overlying the craniectomy defect (Fig. 9), which is even more obvious in the vertical position and can be exacerbated by CSF diversion or significant atrophy of the infarcted territory. The pathophysiology of this syndrome appears to be a mismatch between atmospheric pressure and intracranial pressure, leading to the impairment of cerebral perfusion, venous drainage, and CSF dynamics. The vast majority of cases will significantly improve after cranioplasty [58].

### Quality of life after DC

While the RCTs mentioned above were primarily looking at mortality and functional outcome (i.e., mRS within the first year), data on long-term outcome, quality of life, participation in activities of daily living and work, patient-reported outcomes, opinion of family and caregivers, and depression and anxiety is limited. For DESTINY II, the rates of retrospective consent obtained from survivors, assessment of quality of life (EQ-5D and SF-36 questionnaires), and frequency of major depression were published [31]. Additionally, Rahme and co-workers as well as Woertgen and co-workers provide similar information [59, 60]. In summary, 60–80% of survivors and/or caregivers gave retrospective consent to DC, which indicates an at least acceptable outcome. Quality of life was impaired (mean overall reduction of almost 50%) and major depression occurred frequently (almost 60%), but both to an extent known from survivors of other significant cerebral insults. In a small series published by Vahedi and co-workers, no patient (mean age 41 years) returned to prior employment [61]. Quality of life after DC for supratentorial malignant stroke appears to be acceptable for the majority of patients, and most do not regret having undergone DC despite relevant rates of impairment and depression. However, although Woertgen and co-workers indicate that no difference in quality of life was observed when comparing dominant and non-dominant hemisphere stroke patients, those with aphasia might be underrepresented especially in patient-reported outcomes and could be at higher risk of psychosocial impairment [31, 60].

## Infratentorial malignant ischemic stroke in adults

In comparison with supratentorial stroke, there is a paucity of high-quality clinical studies on the role of DC for cerebellar stroke. The best available evidence comes from one prospective observational study, one retrospective matched case-control study and several case series or case reports.

Jauss and co-workers conducted a prospective multi-center observational study exploring the best medical care, external ventricular drainage, and suboccipital DC [62]. The study design does not allow for direct comparison of treatment options, as the groups are imbalanced with regard to the severity of cerebellar infarction. In the DC cohort, 50% of patients were comatose, surgery was performed at a mean time of 62 h after admission, and poor outcome (mRS > 2) was reported in 35%. Clinical deterioration occurred most commonly at day 3 after stroke onset. No comatose patients were enrolled in the medical treatment arm, preventing direct comparison. The only reasonably comparable subgroups in this study are patients classified as somnolent/stuporous, but no difference in the outcome becomes evident when comparing medical care and DC.

Kim and co-workers published results of a retrospective matched case-control study [63]. The surgical patients were treated with suboccipital DC and additionally 50% received an external ventricular drain (EVD) and 57% a debridement of infarcted tissue. Poor outcome (mRS > 2) was reported in 49% in the medical and 33% in the surgical cohort, indicating a significant benefit of DC.

The largest case series was published by Pfefferkorn and co-workers [17]. They included 57 patients in their monocentric retrospective analysis, of which 82% received an EVD and 56% a debridement of infarcted tissue in addition to DC. Poor outcome (mRS > 2) was observed in 60% of patients and in 76% in the subgroup with additional brainstem infarction. Mortality was 40% and 58%, respectively. Surgical complications occurred in 18% of cases, comprising CSF leaks and meningitis/ventriculitis. Two aspects of this study are worth mentioning: age above 60 years as well as the timing of DC did not appear to influence the outcome. Quality of life (SF-36 questionnaire) was moderately impaired, and 96% of survivors retrospectively consented to suboccipital DC.

Without neurosurgical intervention, a mortality of 80% has been reported for patients with cerebellar infarction who develop brainstem compression [64]. Considering this dismal prognosis, DC is a well-accepted treatment option. Significant infarct volume as defined above, first clinical signs of brainstem compression, or radiological evidence of progressive space-occupying effect are considered indications for DC.

## Supra- and infratentorial malignant ischemic stroke in children

Ischemic stroke in children is very rare, with a reported incidence of 1.2 to 3.6 cases per 100,000 per year, and less than 2% are malignant ischemic strokes [65]. Data on the role of DC in children with ischemic stroke is limited to case reports and small case series, as summarized in Table 3 [65–80]. In the absence of evidence-based recommendations, most authors adduce findings from studies in adults as a reference for treatment decisions in children. However, when reviewing the reported cases (*N* = 28), it seems that DC for supratentorial stroke is performed rather late, as a high proportion (84%) of children had preoperative mydriasis indicating herniation. Nevertheless, the outcome appears to be better than in adults (good outcome reported in 96%), which could either be attributed to brain plasticity and higher potential of recovery in childhood or be explained by reporting bias. In the majority of reported pediatric cases (68%), the cause of cerebral infarction has been identified, such as cardiac, hematological, or infectious conditions.

**Table 3:** Studies on supra- and infratentorial DC in children. Overview of the literature on DC for malignant ischemic stroke in children, with important characteristics of each study or case report

Study	Number	Age	Vascular territory	Etiology/risk factors of malignant infarction	Mydriasis	Timing of DC after onset/admission [h]	Outcome
Supratentorial DC							
Aghakhani [66]	1	11 years	MCA R	Unknown	Unilateral	2	GOS 4
Farooq [67]	1	19 months	MCA L	Unknown	No	36–48	mRS 1
Kirton [64]	1	11 years	MCA R	Cardiac intervention	Unilateral	NA	mRS 3
Lammy [68]	1	16 years	MCA R	Unknown	No	< 24	GOS 5
Lee [69]	1	6 years	MCA R	Cardiac myxoma	Unilateral	NA	mRS 2
Lee [70]	4	23 months (17–33)	MCA R, MCA L, left basal ganglia, NA	Unknown	Unilateral (4)	14 (5–19)	GOS 5 (3), GOS 4 (1)
Leonhardt [52]	1	15 years	MCA R	ICA dissection	NA	12	mRS 2
Montgomery [72]	1	7 years	PCA R	Thrombosed basilar tip aneurysm	Unilateral	24	Good
Rahme [73]	1	7 years	MCA L	Unknown	NA	NA	mRS 3
Ramaswamy [65]	4	18 months (6–36)	MCA+ACA L, MCA+ACA R, MCA L, MCA bilat.	ICA stenosis, postoperative after AVM, and cardiac surgery (2)	Unilateral (4)	28 (8–60)	mRS 1, mRS 2 (2), mRS 3
Shah [74]	3	13.3 years (11–15)	MCA R, MCA L, MCA+ACA L	Fanconi anemia, viral illness	Unilateral (2)	57 (30–72)	mRS 3 (2), mRS 2
Smith [76]	7	7 years (2–14)	MCA R (4), MCA L (3)	Sickle cell disease, ICA occlusion (2), meningitis, cardio-embolic (2), vasculopathy	NA	88 (23–291)	Good (7)
Tan [77]	1	2 years	MCA L	Cardiac surgery	Unilateral	NA	mRS 4
Yamaguchi [78]	1	6 years	MCA L	ICA stenosis	Unilateral	116	Good
Infratentorial DC							
Fischer [80]	1	5 months	Cerebellar infarction L	Unknown	No	NA	Good
Miyata [79]	1	11 years	Cerebellar infarction R	VA stenosis secondary to atlantoaxial subluxation	No	NA	Good
Montgomery [73]	3	8 years (6–10)	PICA+SCA+ AICA R, PCA L+PICA+AICA+SCA bilat., AICA+SCA L+AICA+PCA R	VA dissection, arteriopathy, unknown	No	< 72	Good (3)

**Fig. 10:**
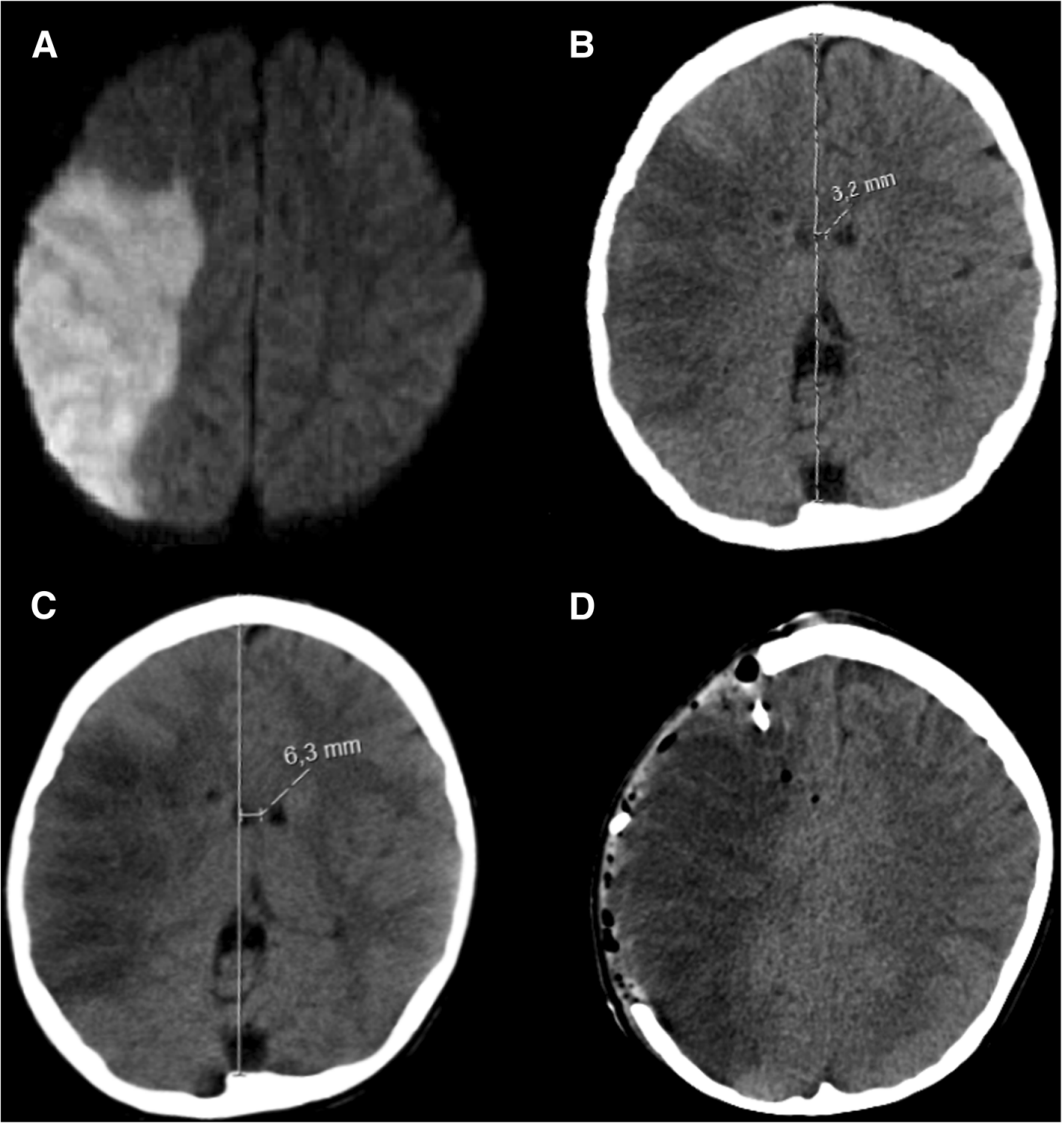
Pediatric ischemic stroke. A representative example of pediatric ischemic stroke in a 6-year-old boy with sickle cell anemia: axial diffusion-weighted MRI sequence (**a**) with increased signal in the right MCA territory, indicating ischemic infarction. Axial CT scan obtained on day 1 after stroke onset (**b**), showing a demarcated infarct with 3.2 mm midline shift. Axial CT scan obtained on day 2 after stroke onset (**c**), revealing a progressive midline shift of 6.3 mm, correlating with neurological deterioration. Axial CT scan after hemicraniectomy and implantation of a right frontal intraparenchymal ICP probe (**d**), with reversal of midline shift

The surgical techniques of hemicraniectomy and suboccipital DC in children are similar to adult patients (Fig. 10). As head growth is accelerated mainly in the first year of life, adapting craniectomy size to age can be neglected beyond infancy and an adequately sized decompression should be achieved in older children as described above. In our monocentric cohort of children undergoing DC at a mean age of 13 years, the mean anterior to posterior diameter of the craniectomy area was 11.7 cm (unpublished data). Importantly, DC in children can lead to significant intraoperative blood loss of up to 50% of estimated blood volume and thus requires adequate preparation by the neurosurgical and anesthesiological team [81].

## Status quo of DC for ischemic stroke: clinical guideline recommendations

The most comprehensive guideline to date on the management of patients with ischemic stroke was published in 2018 by the American Heart Association and American Stroke Association [82]. The guideline recommends early transfer of patients at risk of malignant cerebral edema to a center with neurosurgical expertise. Patient-centered preferences in shared decision-making regarding the interventions and limitations of care should be ascertained at an early stage. With regard to neurosurgical management, the guideline states that in patients ≤ 60 years of age, who deteriorate neurologically (defined as a decrease in the level of consciousness attributed to brain swelling despite medical therapy) within 48 h after MCA infarction, DC with expansion duroplasty is reasonable. In patients > 60 years of age, the same approach may be considered. For patients with cerebellar malignant stroke, the guideline recommends suboccipital DC with expansion duroplasty upon neurological deterioration despite medical therapy, with concurrent EVD insertion to treat obstructive hydrocephalus.

## Future perspectives

The benefit of DC for supratentorial malignant ischemic stroke in adults has been demonstrated by RCTs, as outlined above. However, open questions remain with regard to the long-term effects of chronic physical disability, patient-reported outcomes, depression, and psychosocial impairment as well as the identification of subgroups or specific patient characteristics that might be associated with a greater or lesser benefit from DC. Such information could improve preoperative counseling, as it would enable physicians to more precisely predict the expected disability of individual patients. In times of personalized medicine, better prognostication based on high-quality clinical data, refined imaging, biomarkers, and genetic markers would help identify individuals who benefit from early DC most. Therefore, ongoing research in this field has to be encouraged, although the future role and frequency of DC will be influenced by other treatment modalities: our group has demonstrated that the publication of positive RCTs on DC for stroke has increased annual numbers of DC in the past, but that publication of RCTs on mechanical thrombectomy and subsequent clinical implementation of this revolutionary new modality have already reversed this trend [83]. Despite many negative results from specific targeting of secondary brain injury, this field might offer additional beneficial medical and critical care treatment options in the future [84].

While the efficacy of suboccipital DC in alleviating brainstem compression and thus reducing mortality is well accepted, data is limited on optimal timing and benefit of patient subgroups. Such specific aspects should be analyzed by prospective studies or registries. The same holds true for children, where the evidence base is even more limited.

However, when retrieving current entries from the ClinicalTrials.gov database regarding “decompressive craniectomy,” an apparent shift in clinical research focus becomes obvious: current prospective studies analyze the syndrome of the trephined (NCT03186157) and resorption of autologous bone flaps (NCT02320955). Similar prospective studies are also conducted elsewhere, such as the German Cranial Reconstruction Registry (German Clinical Trials Register ID DRKS00007931) [85]. This shift in focus towards cranioplasty will hopefully provide valuable data to optimize treatment in the aftermath of DC.

## Conclusions

DC is an important treatment option in malignant stroke across all age groups. RCTs of DC for supratentorial malignant stroke confirmed a significant reduction of mortality. This effect is also evident in studies of DC for infratentorial stroke. However, DC renders a relevant proportion of patients with a moderately severe disability. Precise knowledge of the relevant data is therefore crucial in the decision-making process for individual patients. Deciding who is a candidate for early or preemptive surgery and who might benefit from postponing surgery until clear evidence of deterioration evolves can be challenging. An even greater challenge might be to ascertain whether the patient will have acceptable disability and quality of life in his or her presumed perception, based on preoperative predictions. For cerebellar malignant stroke, for malignant stroke in children, and for cranioplasty after DC, the level of evidence is overall lower. As RCTs might not be adequate for certain surgical questions, we emphasize the value of well-designed cohort or case-control studies as well as prospective multi-center registries. Future studies should aim to refine our knowledge and evidence base on DC and should adapt to the paradigm of personalized medicine by more precisely predicting when and how to perform DC in specific patients to achieve optimal outcomes.

## Data Availability

Not applicable.

## Abbreviations

ACA: Anterior cerebral artery
AICA: Anterior inferior cerebellar artery
AVM: Arteriovenous malformation
CSF: Cerebrospinal fluid
CT: Computed tomography
DC: Decompressive craniectomy
EVD: External ventricular drain
ICA: Internal carotid artery
ICP: Intracranial pressure
MCA: Middle cerebral artery
MRI: Magnetic resonance imaging
mRS: Modified Rankin Scale
PCA: Posterior cerebral artery
PICA: Posterior inferior cerebellar artery
RCT: Randomized controlled trials
SCA: Superior cerebellar artery
TBI: Traumatic brain injury
